# Development of a PCR Assay for the Identification of *Salmonella* Thompson

**DOI:** 10.3390/microorganisms14040927

**Published:** 2026-04-20

**Authors:** Dele Ogunremi, Naana Duah, Tianbi Tan, Bei Zhang, Lawrence Goodridge

**Affiliations:** 1 Ottawa Laboratory Fallowfield, Canadian Food Inspection Agency, Ottawa, ON K2J 4S1, Canada; nduah089@uottawa.ca (N.D.); tianbi.tan@inspection.gc.ca (T.T.); bei.zhang@inspection.gc.ca (B.Z.); 2 Department of Food Science, University of Guelph, Guelph, ON N1G 2W1, Canada; goodridl@uoguelph.ca; 3 Department of Biology, University of Ottawa, Ottawa, ON K1N 6N5, Canada; 4 Department of Chemistry, Carleton University, Ottawa, ON K1S 5B6, Canada

**Keywords:** *Salmonella* Thompson, PCR, identification, specific, common serovars, Enteritidis, Typhimurium, Heidelberg

## Abstract

The effective control of foodborne salmonellosis relies on the rapid and reliable detection and identification of the pathogen. Reliable detection tools for identifying the most common *Salmonella* serovars should translate to a considerable alleviation of the health burden attributed to *Salmonella*. We have developed a PCR assay for the rapid identification of colonies of *Salmonella enterica* serovar Thompson, a common serovar. Genomic analyses of publicly available sequences of *Salmonella* Thompson revealed the presence of a unique, Thompson-specific fragment, which we have used to design a pair of oligonucleotides, STho-F and STho-R, for the PCR amplification of an 808 bp DNA fragment. Using crude DNA extracts, the 808 bp fragment was detected in 77 out of 78 isolates of *S.* Thompson (sensitivity = 98.7% *n* = 78 isolates) but not in any of the non-*Salmonella* organisms tested (*n* = 100; 100% specificity) nor in non-Thompson *Salmonella* serovars (*n* = 100; 100% specificity). The sensitivity (inclusivity) and specificity (exclusivity) indices of the PCR assay for *S*. Thompson met the standard regulatory requirements. The Thompson primer pair was compatible with other primers pairs in a multiplex PCR designed for three other common *Salmonella* serovars. Colonies belonging to the Enteritidis serovar (*n* = 100), Heidelberg serovar (*n* = 100), Typhimurium serovar (*n* = 100), and Thompson serovar (*n* = 77) were correctly designated, indicating excellent inclusivity and exclusivity scores for all four *Salmonella* serovars tested in a single multiplex PCR.

## 1. Introduction

*Salmonella* is a common cause of foodborne illnesses and remains a persistent threat to public health and food safety on a global scale. The organism is responsible for a considerable health burden due to high disease prevalence, high rate of hospitalization, and occasional deaths during outbreaks [1]. Over 2600 serovars of *Salmonella* have been identified. However, the number of serovars associated with foodborne illnesses is small, and it is restricted to *Salmonella enterica* subsp. *enterica* [2,3]. Two serovars of this sub-species, namely *Salmonella enterica* serovars Enteritidis and Typhimurium, are consistently the most widely reported among serovars in most areas of the world; however, a few other serovars, including *Salmonella enterica* serovar Thompson, are increasingly responsible for multiple documented foodborne outbreaks in many regions of the world. The outbreaks caused by *S.* Thompson are linked to truly diverse food sources, including produce, poultry spices, seafood, roast beef, fresh cilantro, lettuce, and ready-to-eat foods. Large *S*. Thompson outbreaks, involving dozens to thousands of people, have been attributed to chocolate cake in Korea [4], onions in the United States [5], seafood in the United States [6], smoked salmon in the Netherlands [7], roast beef [8], and fresh cilantro in the United States [9].

The Public Health Agency of Canada conducts annual surveillance of enteric diseases, including *Salmonella* infection, and determines the serovar of each recovered *Salmonella* isolate. For the most recent decade for which reports are available (2014 to 2023), *Salmonella enterica* serovar Thompson has been placed, on average, among the top 10 *Salmonella* serovars recovered from humans in Canada [10,11]. In China, the serovar Thompson is among the top serovars, and it displayed strong antimicrobial resistance against three front-line antimicrobial agents, namely fluoroquinolones (e.g., ciprofloxacin), third-generation cephalosporins (e.g., cefotaxime and ceftriaxone), and macrolides (e.g., azithromycin), making prompt identification of the serovar imperative during food safety investigations [12]. Recent large outbreaks of the serovar Thompson in the US [5,6] and Korea [4] have renewed focus on the serovar since its first description in 1920 and have prompted new efforts to develop a rapid detection method for regulatory use [13].

Commercial PCR assays are available for the detection of *Salmonella* and for identifying the two most common serovars, namely Enteritidis and Typhimurium. At least three commercial offers are currently available for the detection of both serovars Typhimurium and Enteritidis (Neogen, https://www.neogen.com, ThermoFisher, https://www.thermofisher.com/ and Hygiena, https://www.hygiena.com/), while another company offers a customizable PCR assay able to detect four serovars (Genopathx, www.quantipath.bio). None of the assays detect the serovar Thompson. Previously, we had developed a multiplex PCR assay for the identification of the *Salmonella* organisms and for identifying the colonies belonging to serovars Enteritidis and Typhimurium [14]. We subsequently included the serovar Heidelberg in the multiplex PCR to allow for the identification of the three most common serovars of *Salmonella* in Canada [15].

In the present study, we identified a unique DNA fragment in the genome of *S.* Thompson and designed a pair of oligonucleotide primers for the use in developing a specific PCR assay based on the amplification of a unique 808 bp fragment of *S*. Thompson. We have now added the new primer pair to our existing multiplex PCR to develop a new assay for the identification of colonies belonging to four *Salmonella* serovars, including Thompson.

## 2. Materials and Methods

### 2.1. Bacterial Strains

All bacterial isolates tested in this study were retrieved from the CFIA National Food Microbiology Bank and consisted of organisms originally isolated from food, the environment, and other sources, including one human isolate. A total of 278 bacterial strains were tested and summarized in Table 1. They belonged to three groups, namely: serovar Thompson (*n* = 78), *Salmonella* strains of serovars other than the serovar Thompson (*n* = 100), and non-*Salmonella* bacterial organisms (*n* = 100). The available detailed descriptions of each organism are presented in Supplementary Table S1.

### 2.2. Identification of a Salmonella Thompson-Specific Chromosomal Region and the Development of PCR Primers

To design PCR primers that are specific for the serovar Thompson, it was necessary to define a chromosomal region present only in *S.* Thompson but absent in all the other *Salmonella* serovars and in other bacterial organisms. The complete genome sequence of *Salmonella enterica* subsp. *enterica* serovar Thompson strain RM6836 was retrieved from the National Center for Biotechnology Information (NCBI) database (GenBank accession number CP006717, size 4,707,648 bp). Regions of RMS6836 unique to the serovar but absent in other *Salmonella* organisms were analyzed by carrying out a Basic Local Alignment Search Tool (BLAST v2.2.26) analysis of the genome against all *Salmonella* genomes in the GenBank (https://blast.ncbi.nlm.nih.gov/Blast.cgi, accessed on 10 August 2021). The database core_nt was searched with the Megablast option, which was optimized for highly similar sequences, using default standard settings of the expected thresholds of 0.05 and word size of 28. The searches were done reiteratively to ensure that the region obtained at the end was unique to *S.* Thompson. In one step, the Thompson chromosome was searched against all *Salmonella* but excluding serovar Thompson genomes. Any region showing uniqueness was excised from the genome sequence with the aid of the Clone Manager software (Sci-ED software version 9, Cary, NC, USA), and re-submitted for a BLAST analysis; the steps were repeated until very precise coordinates of unique regions were determined. The resultant Thompson DNA fragment was analyzed for uniqueness through a BLAST analysis against all bacteria but excluding all *Salmonella* sequences to evaluate specificity. In the end, a sequence contained within the *S.* Thompson genome and present in all Thompson strains in the GenBank but not found in any other *Salmonella* serovar nor in any non-*Salmonella* genomes was targeted for primer design. The Thompson-specific chromosomal fragment was transferred into the Clone Manager software for the design of the primer pair with a specification to target an amplicon size of approximately 800 bp to ensure that it would be easily discernible among the multiple bands generated in a multiplex PCR containing other targets, including Enteritidis and Typhimurium [14]. Primer criteria included the GC range of 50–60%, Tm range of 55–80 °C, annealing temperature of 55 °C, stability of 1.2 kcal, and 3’ dimers of <3. The forward and reverse oligonucleotide primers were designated as ThoF and ThoR, respectively.

### 2.3. DNA Template Extraction

The DNA samples used as templates for PCR were prepared as crude bacterial extracts. The starting material was either bacteria retrieved from cold storage or grown on Brain Heart Infusion (BHI) agar plate through streaking, followed by an overnight incubation at 37 °C. A loop of bacteria from a slightly thawed vial or a single colony-forming unit (CFU) from a BHI agar plate was placed in a 1.5 mL Eppendorf microfuge tube containing 50 µL of water. The microfuge tube was heated in a boiling water bath for 10 min and then centrifuged at 18,200× *g* for 2 min. The supernatant, which contains DNA, was diluted 1/10 by taking 5 µL of it and adding it to a new Eppendorf microfuge containing 45 µL of distilled water, and it was subsequently used as the DNA template for PCR amplification. To evaluate the analytical sensitivity of the assay, we extracted pure DNA from an overnight culture of *S.* Thompson in BHI media at 37 °C using the Wizard Genomic DNA purification kit (Promega, Madison, WI, USA). DNA quality was assessed using the NanoDrop 2000 UV-Vis (Thermo Fisher Scientific, Waltham, MA, USA) to determine A260/280/230 values, and concentration was measured using the Qubit^®^ Fluorimeter (Thermo Fisher Scientific, Waltham, MA, USA).

### 2.4. PCR Assay for the Detection of Salmonella Thompson

To test the ability of the ThoF and *ThoR* primer pair to recognize and amplify the Thompson-specific fragment, we developed a PCR assay under conditions that are expected to be optimal for the amplification of the fragment. As an internal control for confirming the presence of bacterial DNA template in the reaction, we included a second primer pair, namely E334F [16] and 295526R [14], designed to amplify a 1036 bp of bacterial 16S rDNA sequence in the reaction (Table 2). The fragment served as an internal control to prove that failure to detect a Thompson-specific fragment could not have occurred due to a technical error in which bacterial DNA was inadvertently omitted. All primers in this study were manufactured and desalted by Integrated DNA Technologies (Coralville, IA, USA). PCR amplification reagents for *S.* Thompson and for the internal control were added in a 50 µL reaction volume and consisted of the bacterial DNA template (5 µL of diluted crude extract), *Taq* DNA polymerase (1.5 U), deoxynucleoside triphosphate mix (300 µM), MgCl_2_ (2 mM; Thermo Fisher Scientific, Mississauga, ON, Canada), and the mixture of two pairs of primers, namely ThoF/ThoR and E33F/29552R, and sterile distilled water. The PCR cycling conditions were as follows: denaturation at 94 °C for 5 min, followed by 30 cycles of denaturation for 1 min at 94 °C, annealing at 65 °C for 1 min, and extension at 72 °C for 1 min. The final extension step proceeded for another 10 min. The PCR cycling conditions were identical to previously validated criteria for the serovars Enteritidis and Typhimurium [14]. Following the PCR, the amplified DNA fragments were analyzed on the QIAxcel advanced automated electrophoresis system (Qiagen, Mississauga, ON, Canada). Fragment electrophoresis was carried out by loading the PCR-amplified products on a QIAxcel calibrated cartridge (QIAxcel DNA Screening kit, Qiagen, Hilden, Germany), and the analysis was performed in the presence of alignment markers, following the manufacturers recommendations.

For the determination of the limit of detection, a 5-fold titration of purified DNA of *S.* Thompson (see Section 2.3 above) was used in place of a crude bacterial extract at 1.895, 0.379, 0.076, 0.015, 0.003, and 0.001 ng/μL of pure DNA. Then, PCR was carried out and the products were analyzed as described above.

### 2.5. PCR Assay to Test for Primer Compatibility in a Multiplex PCR Designed to Detect Salmonella and Identify the Presence of the Enteritidis, Typhimurium, Heidelberg, and Thompson Serovars

In a distinct set of PCR analyses aimed at simultaneously detecting *Salmonella* and identifying four Salmonella serovars, another three pairs of primers previously developed and shown to amplify specific serovars were added to the reaction to check for primer compatibility and the lack of interference in the multiplex PCR format. The primer pairs were Heidelberg (Heid5F/R), Typhimurium (STM4497F/R), Enteritidis (SE1472298-2F/R), invasin A (*Salmonella invAF/R*), and iron glucosyl transferase (iroBF/R). The primers for *STM4497*, *invA* and *iroB* loci were previously described by Shanmugasundaram et al. [17]. The SE1472298-2 primers were modified from the 293 bp fragment described by Agron et al. [18] to generate a 612 bp fragment of the SE1472298-2 gene [14]. The PCR cycling condition of the multiplex PCR for the identification of multiple serovars was identical to the one used for the serovar Thompson.

**Table 2:** List of oligonucleotide primer pairs used in the multiplex polymerase chain reaction assay for the identification of the *Salmonella* genus and four *Salmonella* serovars.

Serovar/Specificity	Primers	Sequence 5′-3′	Amplicon Size (bp)	References *
*Salmonella* Thompson	ThoF	TGCTTGGGTTCCGCATGGAG	808	This study
	ThoR	AGTCCCGCGATATCACGCAG		
*Salmonella* Enteritidis	SE1472298-2F	CTTGGAGAGCTGCGCTAAAG	612	[14,18]
	SE1472298-2R	TAAGGCACCTCTCAACACTG		
*Salmonella* Typhimurium	STM4497F	GGAATCAATGCCCGCCAATG	523	[17]
	STM4497R	CGTGCTTGAATACCGCCTGTC		
*Salmonella* Heidelberg	Heid5F	GCGTTGCTTCCCTTATGTTG	124	[15]
	Heid5R	GCGTTGAGTGCTGAAATCTG		
*Salmonella iroB*	IroB-F	GAACGTACACCTGATCGCAAG	309	[17]
	IroB-R	GCACCCTGGCCAAAGACTATC		
*Salmonella invA*	InvA-F	ATCAGTACCATCGTCTTATCTTGAT	211	[17]
	InvA-R	TCTGTTTACCGGGCATACCAT		
Universal RNA	E334-F	CCAGACTCCTACGGGAGGCAG	1026	[14,16]
	295526-R	ACGATTACTAGCGATTCCG		

* The cited reference represents the original description of primers.

## 3. Results

### 3.1. Identification of a Unique 808 bp Fragment of Salmonella Thompson

We compared the genome of the reference *S.* Thompson strain RM6836 with the other Thompson genomes in the GenBank, as well as with other bacteria and archaeal organisms, and found a unique, Thompson-specific DNA fragment coding for a hypothetical protein consisting of 148 amino acids, located between 355,133 and 355,575 bp in the reference genome. We also noticed that the non-coding nucleotide sequences flanking the gene on both sides were also specific for the serovar Thompson. Given the need to amplify an appropriately-sized DNA fragment suitable for future integration into a multiplex PCR format, we proceeded to design forward (ThoF) and reverse (ThoR) PCR primers suitable for the amplification of a unique 808 bp unique fragment corresponding to 354,812–355,619 bp location in our reference genome.

### 3.2. PCR Assay for the Detection of Salmonella Thompson

**Figure 1:**
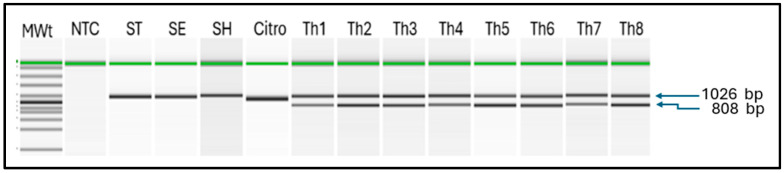
Polymerase chain reaction (PCR) detection of *Salmonella enterica* serovar Thompson.

We developed and assessed our PCR protocol for *S.* Thompson, which included both the primer pair specific to the *S.* Thompson serovar (ThoF and ThoR) and the ribosomal DNA internal control (E334F and 295526R). DNA templates were extracted from 78 isolates of *S.* Thompson and were able to amplify the 808 bp Thompson-specific fragments in 77 isolates (sensitivity = 98.7%). When the assay was applied to 100 bacterial strains belonging to the *Salmonella* serovars other than Thompson, the 808 bp fragment was not observed in any of the samples (Table 1, Figure 1). Similarly, the 808 bp fragment was not detectable in any of the 100 bacterial strains belonging to other genera, including *Citrobacter* (*n* = 13), *Proteus* (*n* = 15), and *Escherichia coli* (*n* = 11), which are sometimes confused with *Salmonella* and others (*n* = 61; Supplementary Table S1). When we replaced the crude bacterial extract with titrated, purified DNA (OD260/280 = 1.9, OD260/230 = 2.1, 37.9 ng/μL) as the PCR template, we determined the lower limit of detection of *S.* Thompson DNA in the PCR assay to be 15 pg/μL (Supplementary Figure S1).

Electrophoretic analysis of PCR amplicons using the QIAxcel system identified the specific 808 bp fragment of *S.* Thompson (samples Th1–8). The multiplex assay detected serovar-Heidelberg-specific 124 bp fragment (SH), serovar-Typhimurium-specific 523 bp fragment (ST), serovar-Enteritidis-specific 612 bp fragment (SE), as well as generic 211 bp fragment of the *invA* gene and the generic 309 bp fragment of *iroB* gene which are prevalent among the *Salmonella* serovars. The universal bacterial band of 1026 bp was detected in all bacteria, including *Citrobacter freundii* (control).

Key: MWt = molecular weight ladder, NTC = negative control (water), ST = *Salmonella* Typhimurium, SE = *Salmonella* Enteritidis, SH = *Salmonella* Heidelberg, Citro = *Citrobacter freundii,* Th1 = *S.* Thompson C94800385, Th2 = *S.* Thompson DART2001-70, Th3 = *S*. Thompson LEV 7303 SA 20034741, Th4 = *S*. Thompson SARB #62 CDC B2637, Th5 = *S*. Thompson S-MBS0108A, Th6 = *S*. Thompson S-MBS7974A, Th7 = *S*. Thompson 08D021 13-6, Th8 = *S*. Thompson 265-4.

### 3.3. Multiplex PCR Assay for the Detection of Salmonella—Thompson, Enteritidis, Typhimurium, and Heidelberg Serovars

**Figure 2:**
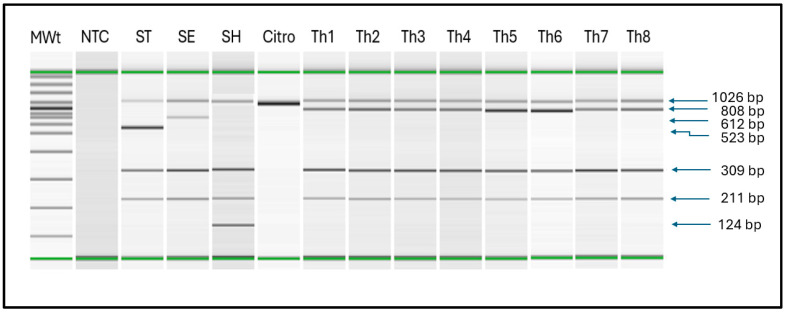
Multiplex polymerase chain reaction (mPCR) detection of *Salmonella enterica* serovar Thompson in an assay that included the use of pairs of generic and serovar-specific *Salmonella* primers.

We have previously described a PCR assay that identified colonies belonging to the Enteritidis and Typhimurium serovars [14]. Subsequently, we developed a PCR method for detecting Heidelberg serovar and incorporated it into a multiplex PCR assay [15]. We have now included the primer pair for serovar Thompson, described in this communication, in the multiplex PCR to detect all four serovars without changing the PCR cycling conditions described above. Upon assessing all DNA extracts from the bacterial strains (Table 1), the multiplex PCR amplified the 808 bp fragment in 77 out of 78 strains of Thompson, reproducing the observations in Section 3.1. As expected, the Thompson-specific fragment was not amplified in the DNA extracts prepared from the other *Salmonella* serovars, which are distinct from the serovar Thompson, nor among the non-*Salmonella* organisms (Figure 2, Supplementary Tables S2 and S3). The results were completely identical to those shown in Table 3 for the Thompson-specific assay, indicating the lack of any interference. There was also no incompatibility problems among the primers. Thus, the multiplex PCR displayed a sensitivity of 98.7% and specificity of 100% in this study.

## 4. Discussion

For rapid identification of *S.* Thompson colonies, we have developed and validated a cost-effective PCR assay that can be applied to crude bacterial DNA extracts, obviating the need to purify genomic DNA and thereby save on costs and time. The assay takes 3 h to complete, and the estimated cost is $6.50 per sample, excluding labour and equipment. The quality metrics of the assay, including excellent specificity (100%) and extremely high sensitivity (98.7%), meet the thresholds for acceptance as a standard test for regulatory use. Our assay compares well and shows distinct strengths and advantages over the efforts of another group to develop a real-time, digital PCR assay for *S.* Thompson. Yang et al. [19] described pairs of specific primers for the amplification of each of 60 serovars of *Salmonella*, including the Thompson serovar (126 bp). The group went on to adapt the Thompson-specific target, identified as the *hsdS* gene, to a digital PCR format [13]. For colony identification, the platforms showed high analytical sensitivity of 10 and 100 CFU/mL for digital and real-time PCR, respectively, and excellent specificity (*n* = 95 strains). However, there was no information on the diagnostic sensitivity of the assay because only five strains of *S.* Thompson were tested. The digital PCR procedure required expensive equipment beyond the reach of many testing laboratories, and the procedure is known to be time consuming. More importantly, the results of the assay become unreliable if a high number of CFU is tested because of the existence of an upper threshold above which the assay does not work. Both assays used purified genomic DNA but did not test against crude bacterial extracts, which we have done in our study. We used a widely available automated DNA electrophoresis procedure, i.e., QIAxcel, which may nevertheless be unaffordable by laboratories in resource limiting settings. However, we were able to demonstrate identical DNA fragment separation results by comparing the QIAxcel with the inexpensive, submarine agarose electrophoresis in a double blinded test design.

Our assay for identifying bacterial colonies of *S.* Thompson is rapid, accurate, cost-effective, and, to ensure its validity, it has been tested against an adequate number of targets for both sensitivity and specificity. The choice of bacteria selected for specificity included those that could be confused with *Salmonella* during the standard isolation procedure because of similar characteristics such as *Citrobacter* and *Proteus* spp. We tested the PCR against 78 strains of the serovar Thompson, and, apart from one strain that tested negative, the remaining 77 strains were positive. We carried out a further evaluation of the negative strain due to the unexpected result and specifically wanted to know if its designation as Thompson at the time of sample receipt in our laboratory was accurate. To that end, we extracted genomic DNA from the organism and carried out whole genome sequencing of pure DNA extract using the GridION instrument to obtain raw genome sequence reads (Oxford Nanopore Technologies, Oxford, UK). The analysis of the DNA sequence using the *Salmonella* In Silico Typing Resource (SISTR [20]) confirmed that the organism belonged to the serovar Thompson. The 808 bp fragment was missing in the raw reads, as well as in the assembled genome, which confirmed the false-negative result for this sole sample. Nevertheless, our assay shows a remarkably high sensitivity and an excellent specificity, meeting the requirements for a standardized colony identification protocol.

As part of food safety testing, the isolation of a suspect bacterial colony on an agar plate is necessarily followed by a process of laboratory confirmation. Traditionally, a combination of procedures such as biochemical tests, agglutination procedures using antisera, and other methods are used to confirm the presence of *Salmonella*, but the procedures are time-consuming and labour-intensive, and the results can sometimes be ambiguous. Instead, a highly specific PCR procedure could provide a rapid, accurate and cost-effective confirmatory result for species and sub-species designation. For the genus *Salmonella*, sub-species designation starting from the serovar level is an important requirement for food safety investigations. Given the total number of serovars at over 2659, developing a PCR assay for each serovar is daunting and impractical, especially because not all serovars are commonly implicated in foodborne outbreaks. All members of the genus *Salmonella* are considered infective to vertebrate hosts, although a limited number, perhaps some 200 serovars, are associated with bacteriemia, disease, or mortality in Canada [21]. Surveillance data on enteric diseases in humans show that only a handful of serovars are responsible for most of the reported human infections: over 80% of reported salmonellosis cases are attributed to 20 serovars [22]. In the United States, 70% of human isolates belonged to 20 serovars, and 98% of infections were attributed to 100 serovars [23]. Similarly, foodborne diseases data in Canada and the US show that only a restricted number of serovars are associated with outbreaks. However, every now and then, a rare serovar emerges as a cause of a widespread, and sometimes very severe, foodborne outbreak. The serovar Adjame, which was reported in London, England in 2017 [24], and the serovar Soahanina, which was reported in Canada and the United States in 2023, originating from cantaloupe imported from Mexico [25], are examples of rare serovars causing notable outbreaks. Unambiguously, year-to-year outbreak data show the consistent trend of the dominance of a few serovars; the serovar Thompson is one of the most prevalent, dominant serovars [10,11].

Although the assay described in this communication is intended for colony identification, there is a possibility of adapting it for a direct detection of contaminants in food and thereby providing a battery of options and versatility for food safety analyses. We propose the use of an enrichment step to ensure the number of bacteria in low-dose contaminated food reach a level detectable by our multiplex PCR. The rapid and accurate detection of *Salmonella* in food is a crucial step towards effective control of human infections, but it is constrained by the small numbers of the organisms commonly present in food. When the cold chain is preserved, *Salmonella* does not grow or grow very slowly and can escape detection. The infective dose of *Salmonella* has only been studied in a few serovars. Teunis [26] estimated that as few as 11 CFUs of *Salmonella* Enteritidis in an egg dish resulted in symptoms in 55% of people who ate the contaminated food (i.e., 198 out of 363). Detection procedures that allow rapid amplification of DNA targets, such as PCR, are useful in detecting contamination and, more importantly, can be completed within a short turnaround time. They also have the advantage of not requiring a prior isolation of the organism, which often takes days to accomplish. On the other hand, certain food matrices may contain materials that could inhibit the polymerase-based DNA amplification procedure and render the method unreliable because of diminished sensitivity. Metagenomics-based detection of pathogens is a newly emerging method that can overcome matrix-induced inhibition observed for PCR methods by employing sequencing to generate an incredibly large amount of information on the contaminating microbial population [27,28].

Even then, the isolation of an organism is still considered important for regulatory purposes because of the need to assess and confirm risk to the consumer. The detection of nucleic acid of a pathogen from food does not implicitly indicate that the consumer is at risk. The presence of live pathogens removes any doubt about the potential of contaminated food to harm the consumer. Consequently, a standardized, quality-assured procedure for the isolation of a microbial contaminant remains one of the most widely used approaches for detecting food contamination in both resource-limiting and resource-sufficient areas of the world. Our validated method for identifying *Salmonella* colonies and for determining if they belong to four of the common serovars should help improve the cost and speed of delivery of *Salmonella* testing.

## Figures and Tables

**Table 1:** *Salmonella* isolates tested in this study.

Bacterial Organism	Number of Organisms (*n*)
*Salmonella* serovar Thompson	78
*Salmonella* serovars other than serovar Thompson	100
Non-*Salmonella* organisms	100

**Table 3:** Polymerase chain reaction (PCR) assay for the detection of the 808 bp *Salmonella* Thompson-specific fragment in bacteria strains.

Bacterial Strain	No. Tested	Positive	Negative	% Sensitivity	% Specificity
*Salmonella* Thompson	78	77	1	98.7	-
*Salmonella* serovars other than Thompson	100	0	100	-	100
Non-*Salmonella*	100	0	100	-	100

## Data Availability

The original contributions presented in this study are included in the article/Supplementary Materials. Further inquiries can be directed at the corresponding author.

## Supplementary Materials

The following supporting information can be downloaded at: https://www.mdpi.com/article/10.3390/microorganisms14040927/s1, Figure S1: Limit of detection of *Salmonella enterica* serovar Thompson polymerase chain reaction assay. Table S1: *Salmonella enterica* serovar Thompson strains. Legend: Strains of Salmonella enterica serovar Thompson (Th1–Th78, *n* = 78) were tested by PCR for *Salmonella* serovar Thompson and were also tested by a multiplex PCR (mPCR) which included additional primers for detecting *Salmonella* serovars Enteritidis, Typhimurium and Heidelberg as well as primers for the genus Salmonella and for a universal bacteria control. Table S2: Non-*Salmonella* organisms. Legend: Strains of non-*Salmonella* bacterial organisms (*n* = 100) were tested by PCR for *Salmonella* serovar Thompson and were also tested by a multiplex PCR (mPCR) which included additional primers for detecting Salmonella serovars Enteritidis, Typhimurium and Heidelberg as well as pri mers for the genus *Salmonella* and for a universal bacteria control. Table S3: *Salmonella enterica* serovars excluding serovar Thompson. Legend: Strains of *Salmonella* organisms belonging to various serovars (*n* = 100) but not the serovar Thompson were tested by PCR for *Salmonella* serovar Thompson and were also tested by a multiplex PCR (mPCR) which included additional primers for detecting Salmonella serovars Enteritidis, Typhimurium and Heidelberg as well as primers for the genus *Salmonella* and for a universal bacteria control.
