# Supplementary figures and images for: Development of a PCR Assay for the Identification of Salmonella Thompson
Source: Microorganisms. 2026 Apr 20;14(4):927. doi: 10.3390/microorganisms14040927 (PMC13118338; doi:10.3390/microorganisms14040927)

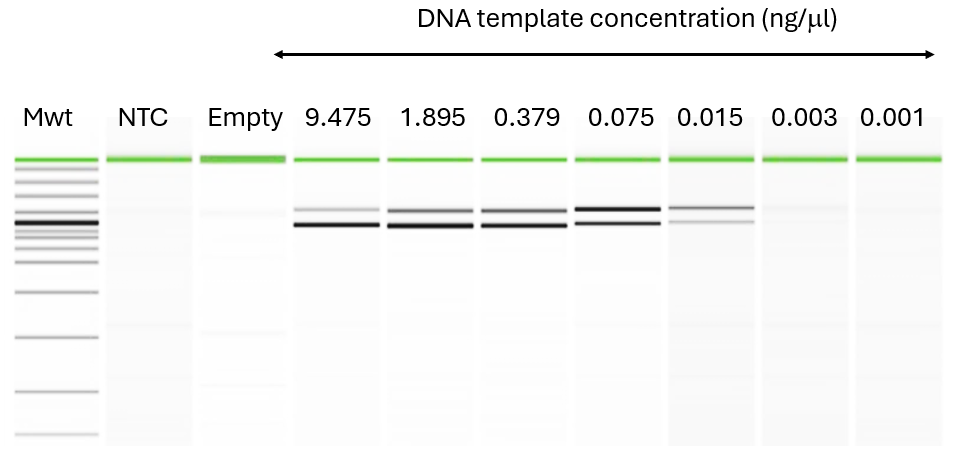

Supplement: Supplementary file 1 [file microorganisms-14-00927-s001.zip › Figure S1.png]
